# HCA (2-Hydroxy-Docosahexaenoic Acid) Induces Apoptosis and Endoplasmic Reticulum Stress in Pancreatic Cancer Cells

**DOI:** 10.3390/ijms23179902

**Published:** 2022-08-31

**Authors:** Roberto Beteta-Göbel, Marc Miralles, Javier Fernández-Díaz, Raquel Rodríguez-Lorca, Manuel Torres, Paula Fernández-García, Pablo V. Escribá, Victoria Lladó

**Affiliations:** 1 Laboratory of Molecular Cell Biomedicine, Department of Biology, University of the Balearic Islands, 07122 Palma de Mallorca, Spain; 2 R&D Department, Laminar Pharmaceuticals, C/Isaac Newton, 07121 Palma de Mallorca, Spain

**Keywords:** HCA, membrane lipid therapy, pancreatic cancer, apoptosis, ER stress

## Abstract

Pancreatic cancer has a high mortality rate due to its aggressive nature and high metastatic rate. When coupled to the difficulties in detecting this type of tumor early and the lack of effective treatments, this cancer is currently one of the most important clinical challenges in the field of oncology. Melitherapy is an innovative therapeutic approach that is based on modifying the composition and structure of cell membranes to treat different diseases, including cancers. In this context, 2-hydroxycervonic acid (HCA) is a melitherapeutic agent developed to combat pancreatic cancer cells, provoking the programmed cell death by apoptosis of these cells by inducing ER stress and triggering the production of ROS species. The efficacy of HCA was demonstrated in vivo, alone and in combination with gemcitabine, using a MIA PaCa-2 cell xenograft model of pancreatic cancer in which no apparent toxicity was evident. HCA is metabolized by α-oxidation to C21:5n-3 (heneicosapentaenoic acid), which in turn also showed anti-proliferative effect in these cells. Given the unmet clinical needs associated with pancreatic cancer, the data presented here suggest that the use of HCA merits further study as a potential therapy for this condition.

## 1. Introduction

It has been estimated that 62,210 new cases of pancreatic cancer will be diagnosed in the United States in 2022, leading to an estimated 49,830 deaths from this disease. Based on these figures, pancreatic cancer will be the fourth highest cause of cancer-related death in the US [1] and the seventh leading cause of cancer death worldwide, making it the most lethal of the common malignancies [2]. In addition, the median survival of pancreatic cancer patients is 10–12 months with treatment and 5–6 months if untreated [1].

Currently, malignant neoplasms of the pancreas are classified according to their differentiation into ductal, acinar, or neuroendocrine cell types, in combination with the macroscopic state of the tumors, solid or cystic. Accordingly, 90% of all pancreatic malignancies correspond to pancreatic ductal adenocarcinomas (PDAC), 5% to pancreatic neuroendocrine tumors, and the remainder to solid-pseudo papillary neoplasms, acinar cell carcinomas, and pancreatoblastomas [3].

The standard treatment for pancreatic cancer involves the use of gemcitabine and FOLFIRINOX, used as adjuvant chemotherapy after surgery as the standard-of-care for resectable tumors. Gemcitabine is a deoxycytidine analogue that inhibits DNA repair and synthesis through its incorporation into DNA [4]. It is generally used alone or in combination with other anti-tumor drugs (erlotinib, cisplatin, fluoropyrimidine) and albumin-bound paclitaxel to enhance patient survival [5]. FOLFIRINOX is a combination of fluorouracil, leucovorin, irinotecan, and oxaliplatin believed to prolong overall survival (OS) beyond that of gemcitabine in patients with metastatic pancreatic cancer, although it is associated with a higher incidence of toxic effects [6]. Despite recent therapeutic advances, the survival associated with pancreatic cancer remains dismal [7].

One therapeutic alternative is melitherapy (Membrane-Lipid Therapy—MLT), an innovative approach to treat disease, including cancer, and it is based on modifying the composition and structure of cell membranes as a means to halt the progress of different pathologies [8,9]. One of the molecules that can be used in melitherapy is HCA (2-hydroxycervonic acid, 2-hydroxydocosahexaenoic acid, C22:6n-3), a novel hydroxyl-derivative of DHA (docosahexaenoic acid). Epidemiological studies have shown that a high intake of fish oil or ω3 polyunsaturated fatty acids (e.g., DHA) reduces the risk of pancreatic cancers, and that DHA has anti-tumorigenic properties in this type of cancer, although no long-term clinical studies are currently available [10]. HCA is an anti-tumor compound that promotes glioma cell death by inducing endoplasmic reticulum (ER) stress and autophagy [11] and also presents pharmacological safety previously demonstrated in zebrafish, *Drosophila melanogaster*, and mice [12,13,14].

Programmed cell death or apoptosis drives the orderly and efficient elimination of cells after they are damaged or in order to achieve specific pattern formation during development [15]. Apoptosis is a complex process involving many signaling pathways and it can be driven in cells by extrinsic or intrinsic pathways. Both these pathways converge to activate effector apoptotic caspases that induce morphological (cytoplasmic cell shrinkage, plasma membrane budding, extracellular exposure of membrane phosphatidylserine, chromatin condensation, and DNA fragmentation) or biochemical changes in the cell [16]. On the one hand, the extrinsic apoptotic pathway is initiated by death receptors at the cell surface, these belonging to the tumor necrosis factor (TNFR) receptor superfamily that are activated by their respective ligands in the TNF protein family [17]. On the other hand, intrinsic apoptosis is mediated by intracellular signals that converge on the mitochondria in response to different stress conditions (irradiation, chemotherapy, etc.) [18]. In precancerous lesions, cells that have damaged DNA can be eliminated by inducing their apoptosis, thereby removing these harmful cells and blocking tumor growth. However, deregulation of this apoptotic process (the hallmarks of cancer) may favor the development and progression of cancer, as well as resistance to drug therapies [19]. Accordingly, therapeutic strategies that restore the sensitivity of cancer cells to apoptosis represent a valid approach in oncology [20].

In many diseases, misfolded proteins accumulate in the endoplasmic reticulum (ER) of different tissues, such as cancer. Upon accumulation of these aberrant proteins, cells activate the unfolded protein response (UPR) to restore proteostasis [21]. However, if this proteostasis of the ER cannot be restored, cell death will be triggered through processes such as apoptosis or autophagy [22]. Autophagy is a means to degrade and recycle dysfunctional cellular components, whereby subcellular membranes are reorganized to sequester parts of the cytoplasm and organelles into autophagosomes, structures that will then be transported to the lysosome to degrade the captured elements [23]. Autophagy is a highly conserved process, tightly controlled by autophagy-related genes (ATGs) such as LC3B [24] and by the autophagosome cargo protein (p62/sequestosome 1), a protein that binds to degradation targets and facilitates selective autophagy [25].

In this study, we have used a series of human pancreatic cancer cells lines as models to evaluate the anti-tumor potential of HCA. We found that this compound induces apoptosis, ER stress, and the production of ROS species in pancreatic cancer cells. Moreover, its metabolization by α-oxidation to C21:5n-3 has been confirmed. Accordingly, HCA would appear to be an interesting candidate drug that could potentially be used to combat pancreatic cancer.

## 2. Results

### 2.1. Efficacy of HCA against Pancreatic Cancer

**Figure 1:**
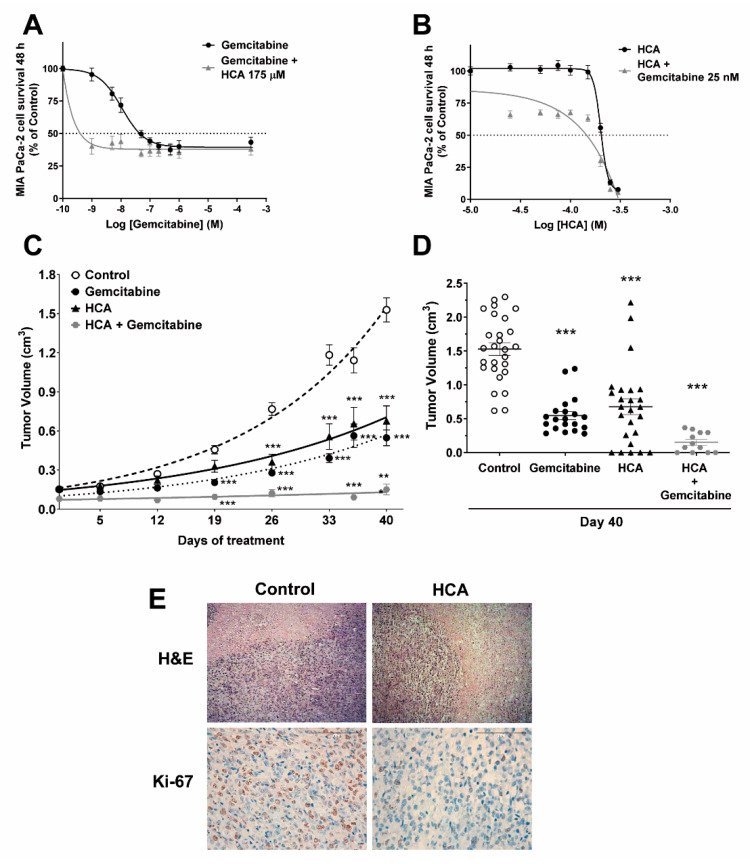
Efficacy of HCA against pancreatic cancer. (**A**) Concentration-dependent inhibition of human pancreatic cell (MIA PaCa-2) growth by gemcitabine and gemcitabine in combination with HCA (175 µM) over 48 h. (**B**) Concentration-dependent inhibition of human pancreatic cell (MIA PaCa-2) growth by HCA and HCA in combination with gemcitabine (25 nM) for 48 h (mean ± SEM from 3 independent experiments performed in quadruplet). (**C**) Effects of the vehicle alone (control), gemcitabine (100 mg/kg, i.p., twice weekly), HCA (200 mg/kg, p.o., daily), or HCA + gemcitabine (100 mg/kg, p.o., daily + 100 mg/kg, i.p., once weekly) on MIA PaCa-2-derived tumor growth in mice over 40 days. (**D**) Tumor volumes after a 40-day treatment (mean ± SEM): control, 27 tumors (*n* = 18, 8 males and 10 females); gemcitabine, 20 tumors (*n* = 15, 4 males and 11 females); HCA, 25 tumors (*n* = 17, 6 males and 11 females); and HCA + gemcitabine, 12 tumors (*n* = 6, 2 males and 4 females). (**E**) Histopathological analysis of the tumors obtained from vehicle and HCA-treated animals (40× magnification). Student’s *t*-test: * < p 0.05, ** *p* < 0.01, *** *p* < 0.001 with respect to the controls. HCA, 2-hydroxycervonic acid; H&E, hematoxylin and eosin; Ki-67, protein proliferation marker.

The efficacy of HCA to impair the growth of pancreatic cancer cells was tested in vitro on a panel of different pancreatic cancer cell lines (MIA PaCa-2, BxPC-3, PANC-1), inhibiting their growth in all cases in a concentration-dependent manner (with an IC_50_ of 205 µM in MIA PaCa-2, 218 µM in BxPC-3, and 232 µM in PANC-1 cells) (Figure 1A,B, Supplementary Figure S1A,B and Supplementary Table S1). In addition, the effect of HCA in combination with gemcitabine, a drug used as standard treatments to combat pancreatic cancer, was also evaluated, assessing HCA at a single concentration below its IC_50_ (175 µM) in combination with increasing concentrations of gemcitabine (0, 1, 5, 10, 50, 100, 200, 500, 1000, 300,000 nM). Likewise, the effect of increasing concentrations of HCA (0, 25, 50, 75, 100, 150, 200, 250, 300 µM) in combination with gemcitabine below its IC_50_ (25 nM) was also tested. In all cases, a stronger shift of the survival curve was observed than with HCA alone, evidence of a synergistic effect when these compounds were combined (Figure 1A,B, Supplementary Figure S1A,B). Indeed, the IC_50_ of gemcitabine in MIA PaCa-2 cells (55 ± 21 nM) was significantly lower in the presence of HCA for 48 h (2 ± 1.9 nM), and similarly, the presence of gemcitabine reduced the IC_50_ of HCA when treating the cells for 48 h (from 205 ± 05 µM to 145 ± 10 µM: Supplementary Table S1). A similar effect was evident on the IC_50_ of gemcitabine when treating BxPC-3 cells for 48 h (180 ± 25 nM), which fell in the presence of HCA (93 ± 40 nM), as did that of HCA (218 ± 06 µM) that fell in the presence of gemcitabine (199 ± 02 µM) when the cells were treated for 48 h (Supplementary Table S1). Interestingly, gemcitabine failed to inhibit the growth of PANC-1 cells, although it was inhibited by HCA at an IC_50_ of 232 ± 16 µM (Supplementary Table S1).

The efficacy of HCA to combat pancreatic cancer was also tested in vivo using the MIA PaCa-2 cell xenograft model. HCA (200 mg/kg, p.o. daily) significantly reduced the xenograft tumor volume (a 56% reduction: Figure 1C,D and Supplementary Figure S1C), as did the drug commonly used as pancreatic cancer treatment, gemcitabine (100 mg/kg, i.p., twice weekly), irrespective of the sex of the mice. Although the mean tumor volume on day 40 of gemcitabine treatment was slightly smaller than when mice were treated with HCA (a 64% reduction: control 1.53 ± 0.09 cm^3^; gemcitabine 0.55 ± 0.06 cm^3^; HCA 0.68 ± 0.12 cm^3^: Figure 1D), unlike HCA, this drug did not completely eliminate any of the tumors. Moreover, the combination of HCA (100 mg/kg, p.o. daily) and gemcitabine (100 mg/kg, i.p. once weekly) proved to be the most effective treatment in vivo, significantly reducing the tumor’s volume (a 90% reduction: control 1.53 ± 0.09 cm^3^; HCA + gemcitabine 0.15 ± 0.04 cm^3^: Figure 1D). During these experiments none of the mice lost more than 10% of their body weight (data not shown), and the oral administration of HCA was well tolerated. Indeed, HCA had been seen not to produce any toxicity in mice in earlier studies at studied doses [11,12,13,14].

An independent analysis of histopathological images failed to identify significant differences between the control animals and those treated with HCA at a dose of 200 mg/kg. The areas of intratumor necrosis, the mitotic count per 10 high-power fields, and the degree of leukocyte infiltration were similar in both cases, and therefore, HCA did not appear to be toxic to these tumor cells (Figure 1E, Supplementary Figure S1). Conversely, a higher proportion of cells with nuclear Ki-67 staining was evident in control tumors (22.50 ± 6.30%) than in those exposed to HCA (17.50 ± 5.95%), indicating that exposure to HCA dampens cell proliferation in these tumors.

### 2.2. HCA Induces Intrinsic and Extrinsic Apoptosis, and ER Stress/UPR Signaling in Pancreatic Cancer Cells

**Figure 2:**
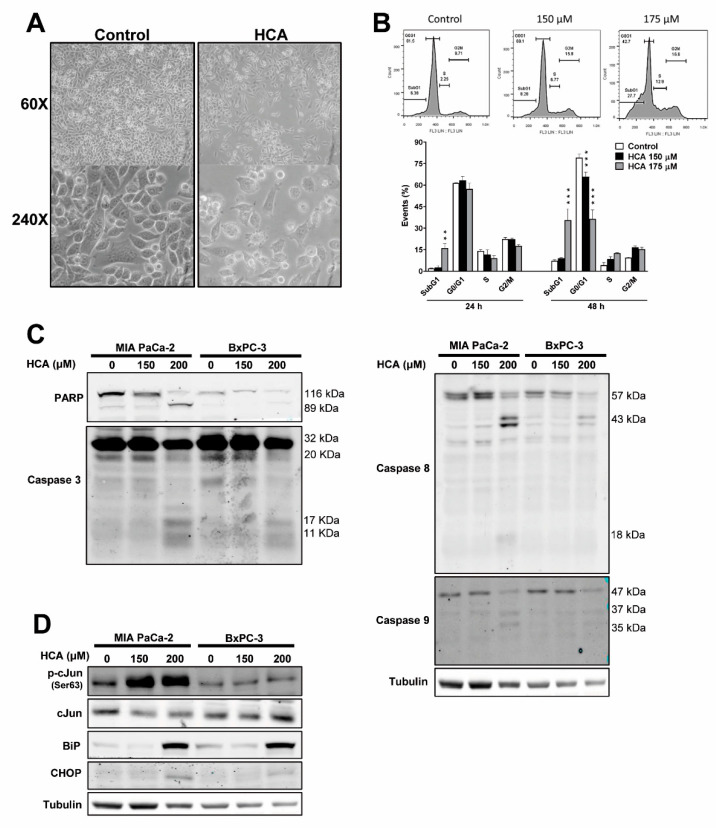
HCA induces intrinsic and extrinsic apoptosis, and ER Stress/UPR signaling, in pancreatic cancer cells. (**A**) Representative microscopy images (60× and 240× magnification) showing the effects of HCA (200 µM for 48 h) on MIA PaCa-2 cells. (**B**) Representative histogram of the cell cycle phases and quantification of the cell populations at different phases of the cell cycle: distribution of MIA PaCa-2 cells incubated with or without HCA (150 or 175 µM) for 24 and 48 h (mean ± SEM of three independent experiments). (**C**) Representative immunoblots of the effect of HCA (150 or 200 µM for 48 h) on the PARP, caspase 3, 8, and 9 proteins in MIA PaCa-2 and BxPC-3 cells, and the amount of tubulin as a loading control. (**D**) Representative immunoblots of the effect of HCA (150 or 200 µM for 48 h) on c-Jun phosphorylation, and on the BiP, CHOP proteins in MIA PaCa-2 and BxPC-3 cells, with tubulin as a loading control. Student’s *t*-test: ** *p* < 0.01, *** *p* < 0.001 relative to the controls. HCA, 2-hydroxycervonic acid; BiP (a.k.a. GRP78), glucose-regulated protein 78; c-Jun, Jun Proto-Oncogene; CHOP (a.k.a. DDIT3), DNA Damage Inducible Transcript 3; PARP, Poly (ADP-Ribose) Polymerase.

Exposure to HCA (200 µM for 48 h) produced morphological changes in MIA PaCa-2 pancreatic cancer cells, which became rounder with some membrane blebbing but without a loss of membrane integrity (Figure 2A). When ethidium bromide-stained MIA PaCa-2 cells were analyzed by flow cytometry, HCA induced a significant increase in the sub-G1 peak, indicative of DNA fragmentation (Figure 2B, Supplementary Figure S2A). This effect of HCA on the sub-G1 peak was concentration-dependent, and it also differed at 24 h (control 1.93 ± 0.25%; HCA 150 µM 2.63 ± 1.25%; HCA 175 µM 16.10 ± 3.10%) and 48 h (control 7.17 ± 0.78%; HCA 150 µM 8.97 ± 0.68%; HCA 175 µM 35.62 ± 7.90%: Figure 2B, Supplementary Figure S2A). In addition, exposure to HCA for 48 h caused a significant concentration-dependent decrease in the proportion of cells at G0/G1 relative to the control cells (control 79.20 ± 2.43%; HCA 150 µM 65.99 ± 3.20%; HCA 175 µM 36.55 ± 6.20%: Figure 2B, Supplementary Figure S2A).

Key proteins involved in apoptosis and in the ER stress/unfolded protein response (UPR) were studied by using Western blot. HCA induced a marked concentration-dependent increase in the proteolytic cleavage of PARP (Poly [ADP-ribose] polymerase) in MIA PaCa-2 and BxPC-3 cells (Figure 2C), a molecular marker of apoptosis [26]. In addition, in both these cell lines there was a concentration-dependent increase in the cleavage of caspase 3, 8, and 9, and hence in the activation of these caspases (Figure 2C). These three caspases are considered to be apoptotic caspases, whereby caspase 3 is the executioner, caspase 8 mediates in the extrinsic or death receptor-dependent pathway, and caspase 9 is involved in the intrinsic or mitochondrial pathway [27]. Thus, HCA appears to activate different apoptotic pathways in pancreatic cancer cells.

Previous studies observed that HCA activated ER/UPR stress signaling pathways in glioma cells [11]. Since RE/UPR stress can trigger cell death, key proteins were studied. HCA (200 µM) appeared to activate ER stress/UPR cascades by augmenting the amounts of BiP (GRP-78 or HSPA5) and CHOP (DDIT3, DNA Damage-Inducible Transcript 3) proteins in both pancreatic cancer cell lines and enhancing c-Jun phosphorylation in Ser63 in MIA PaCa-2 cells (Figure 2D).

### 2.3. HCA Enhances Caspase-3/7 Activity and Mainly Induces Apoptosis of Pancreatic Cancer Cells through the Extrinsic Pathway

**Figure 3:**
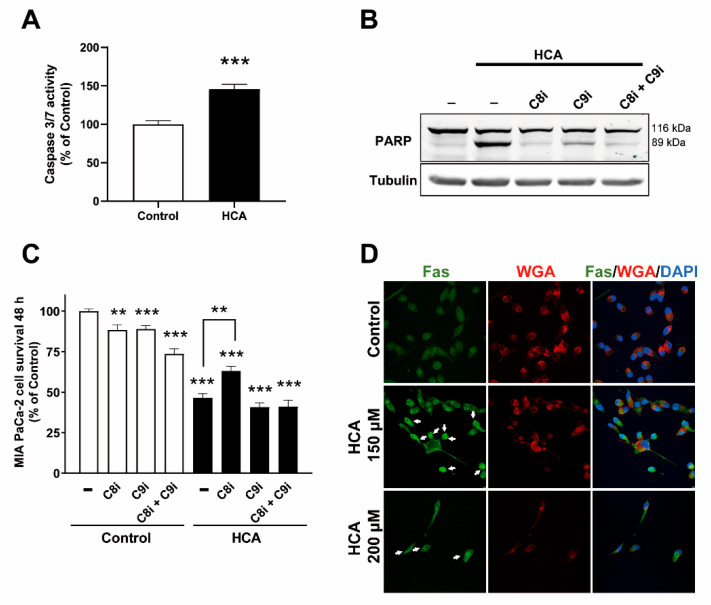
HCA enhances Caspase-3/-7 activity and induces apoptosis in pancreatic cancer cells mainly through the extrinsic pathway. (**A**) Caspase 3/7 activity was measured in MIA PaCa-2 cells as a luminescence read-out (Caspase-3/7 Glo assay) after incubation with HCA (200 µM for 48 h: the bars correspond to the mean ± SEM values of 2 independent experiments performed in triplicate). (**B**) Representative immunoblot of the effects of C8i (inhibitor zIETD-fmk, 50 µM), C9i (inhibitor zLEHD-fmk, 50 µM), and C8i+C9i (zIETD-fmk+zLEHD-fmk, 50 + 50 µM) on PARP proteolysis in MIA PaCa-2 cells treated with HCA for 48 h. MIA PaCa-2 cells were pretreated with the inhibitors for 1.5 h before exposure to HCA (200 µM). (**C**) Effects of C8i, C9i, and C8i+C9i on the survival of MIA PaCa-2 cells treated for 48 h with HCA (the bars correspond to the mean ± SEM of three independent experiments performed in triplicate). (**D**) Labelling of the Fas receptor (green) and Wheat germ agglutinin (red) in MIA PaCa-2 cells cultured for 48 h in the presence or absence of HCA (150 and 200 µM). Capping of the Fas receptors is indicated with arrows. Scale bar 4 µM. Student’s *t*-test: ** *p* < 0.01, *** *p* < 0.001 with respect to the controls. HCA, 2-hydroxycervonic acid; C8i, caspase 8 inhibitor (zIETD-fmk); C9i, caspase 9 inhibitor (zLEHD-fmk); C8i+C9i, caspase 8 inhibitor + caspase 9 inhibitor (zIETD-fmk+zLEHD-fmk); PARP, Poly (ADP-Ribose) Polymerase; WGA, Wheat germ agglutinin; –, the absence of the drug indicated in the figure.

Further investigation of the apoptotic activity induced by HCA (200 µM) in MIA PaCa-2 cells demonstrated enhanced Caspase-3/7 activity (control 100 ± 4.65; HCA 145.80 ± 6.14%: Figure 3A). To examine the specific pathways of apoptosis involved, we exposed these cells to the caspase-8 inhibitor zIETD-fmk that is associated with the extrinsic pathway or the caspase-9 inhibitor zLEHD-fmk associated with the intrinsic pathway. Both these inhibitors dampened the proteolytic cleavage of PARP provoked by HCA (200 µM for 48 h) in MIA PaCa-2 cells, an effect that was more accentuated in the presence of the caspase 8 inhibitor. The simultaneous use of both these inhibitors also inhibited proteolytic cleavage of PARP induced by HCA (Figure 3B, Supplementary Figure S2B). The survival of MIA PaCa-2 cells over 48 h was significantly reduced in the presence of the inhibitors, but the reduction was more than 15% only after the combination of both inhibitors (Control 100 ± 1.37%; C8i 88.3 ± 3.42%; C9i 88.97 ± 2.2%; C8i+C9i 73.54 ± 3.22%: Figure 3C). Indeed, the antiproliferative effect of HCA (200 µM) was impaired by 16.46% in the presence of the caspase 8 inhibitor (HCA 46.61 ± 2.48%; HCA+C8i, 63.07 ± 2.98%: Figure 3C), unlike when these cells were exposed to C9i and a combination of C8i and C9i (HCA 46.61 ± 2.48%; HCA+C8i 40.82 ± 2.56%; HCA+C8i+C9i 41.08 ± 4.02%, Figure 3C). These data suggest that caspase 8 is involved in the apoptosis of MIA PaCa-2. Although, canonically, the activation of the extrinsic pathway (via caspase 8) triggers the induction of the intrinsic pathway, therefore caspase 9 activation, in pancreatic cancer cells [28,29], the activation of caspase 9 by HCA seems to be unrelated to the cell death induction. Hence, HCA would appear to induce apoptosis mainly through the extrinsic or death receptor-dependent pathway. Accordingly, we studied the death receptor, Fas, characterized by a cytoplasmic death domain that allows the receptor to initiate cytotoxic signals when activated [17]. HCA (150 and 200 µM, 48 h) was seen to induce Fas receptor capping in association with alterations to the membrane structure of MIA PaCa-2 cells (Figure 3D). Plasma membrane was labeled with wheat germ, and agglutinin was labeled with Alexa Fluor 594 (WGA). Significantly, there is even evidence that lipid reorganization can induce clustering and activation of death receptors in the absence of ligand (see discussion).

### 2.4. HCA Causes an Increase in Reactive Oxygen Species

**Figure 4:**
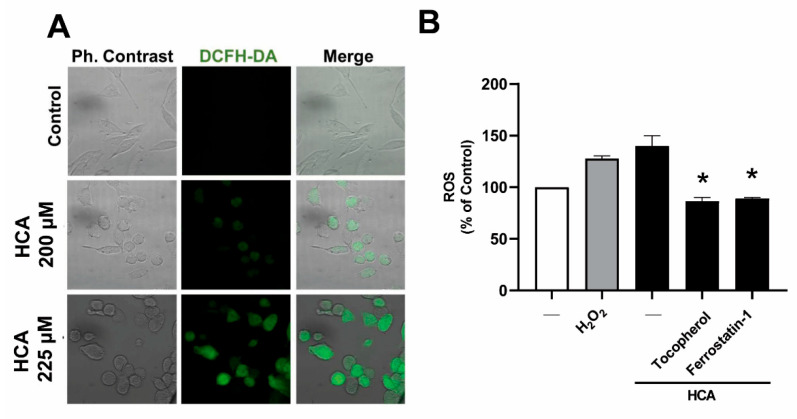
HCA causes an increase in ROS levels. (**A**) Representative confocal images of MIA PaCa-2 cells cultured for 48 h in the presence or absence of HCA (200 and 225 µM). After then, the cells were incubated with 10 µM dichlorofluorescein diacetate for 30 min at 37 °C. ROS species are shown in green. (**B**) Ros levels were measured by flow cytometry. MIA PaCa-2 cells were pretreated with ROS scavengers α-tocopherol (100 µM, Vit. E) or Ferrostatin-1 (1 µM) for 1.5 h before exposure to HCA (200 µM) for 48 h. Positive control H_2_O_2_ (100 µM) (the bars correspond to the mean ± SEM of three independent experiments). Student’s *t*-test: * *p* < 0.05 with respect to the controls. HCA: 2-hydroxycervonic acid. DCFH-DA: 2′, 7′-Dichlorofluorescin diacetate.

Reactive Oxygen Species (ROS) are highly reactive chemicals formed from O_2_, and elevated levels of ROS in the cell can lead to oxidative stress and cell death like apoptosis. For this purpose, the DCFH-DA fluorescent dye was used to detect soluble intracellular ROS. An increase in fluorescence indicative of an increase in ROS was observed in MIA PaCa-2 cells exposed to HCA (200 µM or 225 µM) for 48 h, the intensity of which was concentration dependent (Figure 4A). On the other hand, it was observed by flow cytometry that using ROS scavengers α-tocopherol or Ferrostatin-1 abolished the increase in ROS levels induced by HCA (Figure 4B).

### 2.5. HCA Metabolization by α-Oxidation Does Not Play a Role in Its Mechanism of Action in Pancreatic Cancer Cells

**Figure 5:**
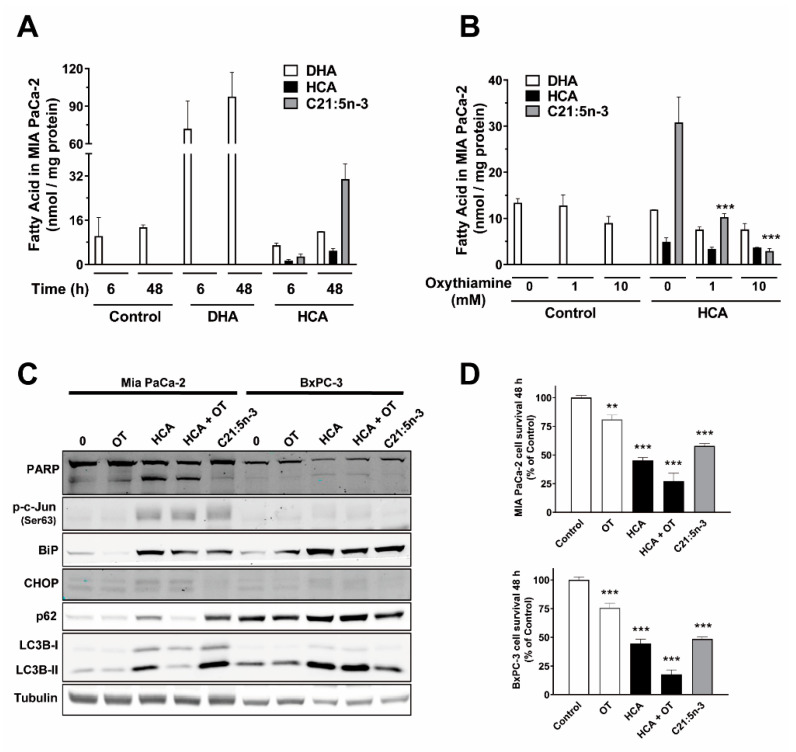
HCA is incorporated into cancer pancreatic cells, and it is metabolized to heneicosapentaenoic acid (C21:5n-3) through α-oxidation. (**A**) Fatty acid levels (DHA, HCA, C21:5n-3) were quantified by GC after HCA or DHA treatment of MIA PaCa-2 cells (the bars correspond to the mean ± SEM values of 3 independent experiments). (**B**) MIA PaCa-2 cells were pretreated with oxythiamine (OT, 1 or 10 mM) for 1.5 h before exposure to HCA (150 µM) for 48 h, and the cell’s lipids were extracted and quantified by GC (the bars correspond to the mean ± SEM values of 3 independent experiments). (**C**) Representative immunoblots of the effect of OT and HCA or C21:5n-3 on ER stress/UPR and autophagy markers in MIA PaCa-2 and BxPC-3 cells. (**D**) Effect of oxythiamine (OT, 1 mM), HCA (200 µM), HCA plus OT (HCA 200 µM plus OT 1 mM) or C21:5n-3 (200 µM) on the survival of MIA PaCa-2 and BxPc-3 cells treated for 48 h (the bars correspond to the mean ± SEM values of 3 independent experiments performed in triplicate). Student’s *t*-test: ** *p* < 0.01, *** *p* < 0.001 with respect to the controls. HCA, 2-hydroxycervonic acid; DHA, docosahexaenoic acid; C21:5n-3, heneicosapentaenoic acid; OT, oxythiamine; BiP (a.k.a. GRP78), glucose-regulated protein 78; c-Jun, Jun Proto-Oncogene; CHOP (a.k.a. DDIT3), DNA Damage Inducible Transcript 3; LC3B, (a.k.a. ATG8F), Microtubule Associated Protein 1 Light Chain 3 Beta; p62 (a.k.a. SQSTM1), Sequestosome 1; PARP, Poly (ADP-Ribose) Polymerase.

The uptake of HCA by the different pancreatic cancer lines was confirmed by gas chromatography, indicating that HCA (150 µM) was taken up by each of the different treated lines (MIA PaCa-2, BxPC-3, and PANC-1) in a time-dependent manner (Figure 5A, Supplementary Figure S3A). HCA accumulated in MIA PaCa-2 cells exposed to HCA in a time-dependent manner (Figure 5A). In addition, the peak corresponding to the HCA’s main metabolite, heneicosapentaenoic acid (C21:5n-3), was observed in the chromatogram from these cells when exposed to HCA in a time-dependent manner but not in those from control cells or those exposed to DHA (docosahexaenoic acid, 100 µM) (Figure 5A, Supplementary Figure S3A), as identified previously in other cells [30,31]. The pathway for the α-oxidation of hydroxylated fatty acids involves a hydroxyacyl CoA lyase (HACL), which in turn depends on thiamine pyrophosphate (TPP) [32]. The metabolization of HCA to C21:5n-3 via α-oxidation was confirmed by the pretreatment of MIA PaCa-2 cells with the α-oxidation inhibitor oxythiamine (OT, 1 mM, or 10 mM), a thiamine analog and competitive inhibitor of both HACL1 and 2. As expected, exposure to OT significantly dampened the formation of C21:5n-3 in cells treated with HCA for 48 h (a 66.92% decrease with OT 1 mM or a 90.5% decrease with OT 10 mM) relative to that formed in the cells treated with HCA alone (from 30.83 ± 5.50 to 10.20 ± 0.75 [1 mM] and 2.93 ± 0.55 nmol/mg protein [10 mM]: Figure 5B). A significant decrease in C21:5n-3 (OT 1 mM 64.31% or 10 mM 82.68%) was also observed in BxPC-3 cells relative to when these cells were treated with HCA alone (from 17.37 ± 0.95 to 6.20 ± 0.98 and 3.01 ± 1.56 nmol/mg protein, respectively: Supplementary Figure S3B), as was also seen in PANC-1 cells (Supplementary Figure S3B).

The proteins involved in apoptosis and ER stress were also studied in MIA PaCa-2 and BxPC-3 cells in presence/absence of HCA α-oxidation inhibition (HCA 200 µM + OT 1 mM) or after treatment of the cells with C21:5n-3 (200 µM) to determine its participation in cell death induction by HCA. Proteolytic cleavage of PARP was observed in cells treated with HCA or HCA+OT but not with C21:5n-3 (Figure 5C). Interestingly, CHOP induction by HCA was not reverted in cells exposed HCA+OT and its levels were not enhanced in those exposed to C21:5n-3 (Figure 5C). Hence, C21:5n-3 would not participate in the apoptosis and CHOP induction by HCA, and the metabolite should trigger mechanisms distinct to those used by the parent molecule. In this sense, there was an increase in the phosphorylation of the ER stress protein c-Jun and in BiP induction under all three conditions, indicating that ER stress/UPR is induced in all cases (HCA, HCA+OT, C21:5n-3: Figure 5C). The status of autophagic flux (autophagic degradation activity) was also studied in these cells. Regarding proteins involved in autophagic flux [33], exposing MIA PaCa-2 cells to HCA and C21:5n-3 increased the conversion of LC3B-I to LC3B-II, and also increases the accumulation of SQSTM1/p62, indicative of enhanced autophagic flux (Figure 5C). Inhibition of α-oxidation (HCA+OT) also appeared to partially inhibit autophagic flux in these cells, but not in BxPC-3 cells. Hence, HCA and C21:5n-3 appear to interact in the ER stress/UPR/autophagy induction by HCA, possibly in a cell specific manner.

The antiproliferative efficacy of C21:5n-3 (200 µM) was studied by assessing how it affects the survival of MIA PaCa-2 and BxPC3 cells over 48 h relative to the controls (untreated). Furthermore, the relevance of the metabolite formation in HCA’s activity was studied by inhibiting its formation with OT addition. The survival of cells was reduced in the different conditions compared to control: treated with HCA (200 µM; 45.21 ± 2.42% in MIA PaCa-2 cells and 44.53 ± 3.66% in BxPC-3), with HCA+OT (HCA 200 µM + OT 1 mM; 27.29 ± 6.71% in MIA PaCa-2 cells and 17.57 ± 3.92%in BxPC-3), and C21:5n-3 alone (200 µM; 57.84 ± 2.08% in MIA PaCa-2 cells and 48.53 ± 1.73% in BxPC-3) (Figure 5D), suggesting that the HCA antitumoral effect is independent of the metabolite formation. The reduction of the survival due to treatment with OT alone (1 mM) was less than 25% (cell survival of 80.94 ± 3.88% in MIA PaCa-2 and 75.69 ± 4.02% in BxPC-3 cells; Figure 5D), taking into account the in vitro and in vivo antitumor effect described previously [34]. In summary, C21:5n-3 has an anti-proliferative effect and the combination of HCA+OT has an additive effect in these conditions and on the cells studied.

Although our results point out to a different mechanism of action for the metabolite than the parent molecule as an antitumoral drug in pancreatic cancer cells, C21:5n-3 is still the main metabolite of HCA, and its presence can be detected in both cells and animals after HCA administration [11,30]. In that context, its safety was assessed following the Irwin test. For that, healthy CD-1 mice were administered a single dose of C21:5n-3 (0, 50, 100, 200, 200, 400, 400, 800, 1000, and 1500 mg/kg: *n* = 3 mice per dose) and its effect on survival was evaluated up to 24 h after C21:5n-3 administration, while its influence on behavioral parameters was evaluated 1 h after administration using the Irwin test [35]. In terms of survival, none of the mice died in the study period at any of the doses administered, and thus, the highest dose of 1500 mg/kg was established as the maximum tolerated dose (MTD). Consequently, no minimum lethal dose (MLD) or lethal dose 50 (LD_50_) could be defined (Supplementary Figure S3). The Irwin test evaluates and quantifies the behavioral and physiological state of the mice in response to a drug and it is comprised of a battery of assays grouped into three major areas: behavioral studies (alertness, grooming, irritability), neurological studies (tremors, convulsions, ataxia), and studies related to the autonomic nervous system (salivation, hypothermia, mydriasis). In no cases were changes observed in the parameters evaluated during the test period (24 h after administration) relative to the control animals, ruling out acute toxic effects of the treatment (Supplementary Figure S3).

## 3. Discussion

Pancreatic cancer is an uncommon but often rapidly lethal malignancy (the seventh most common cause of cancer death) [2]. Currently, there are different first-in-line treatments for pancreatic cancer, involving the administration of gemcitabine and/or FOLFIRINOX (a multidrug combination recommended for metastatic pancreatic cancer), as well as other available treatments. Treatment with FOLFIRINOX has been associated with enhanced patient survival but also with increased toxicity relative to treatment with gemcitabine [36]. Modifications to gemcitabine are currently being studied to improve its effects, such as the covalent binding of gemcitabine to cardiolipin or gemcitabine peptide-based conjugates that enhance its incorporation into cells and improve its effects as an anti-cancer therapy [37,38]. The high mortality and aggressive nature of pancreatic cancer, the challenges of early detection and consequently, their high rate of metastasis, in conjunction with a lack of effective treatments to control the tumor if surgical resection fails, are together responsible for the clinical significance of pancreatic cancers. The present study shows that the hydroxylated fatty acid HCA shows efficacy against cellular and animal models of human pancreatic cancer, inducing cell death by apoptosis independently of its metabolization into C21:5n-3 fatty acid.

Here we studied a rationally designed compound, HCA, as a potential drug to combat pancreatic cancer, having already demonstrated its potential anti-tumor effect against glioma by inducing autophagic cell death [11]. This compound is a derivative of DHA, with a hydroxyl moiety on the α-carbon of DHA. HCA is a molecule developed in the context of melitherapy, a new therapeutic strategy that focuses on modifying the composition and structure of cell membranes as a means to treat different pathologies [8,9,39]. Other molecules designed following this rationale are currently being tested in clinical trials in humans, 2OHOA or 2-hydroxyoleic acid for the treatment of glioma (ClinicalTrials.gov Identifiers: NCT01792310, NCT03867123, NCT04250922, and NCT04299191) and ABTL0812 for the treatment of metastatic pancreatic [40], endometrial and squamous non-small cell lung cancers (ClinicalTrials.gov Identifiers: NCT02201823, NCT04431258, NCT03366480, and NCT03417921).

The data obtained here demonstrate the potential of HCA to treat pancreatic cancer cells. First, HCA inhibited the growth of different pancreatic cancer tumor cell lines, which was further enhanced when administered in combination with gemcitabine, a standard treatment that significantly reduced the IC_50_. Second, the efficacy of HCA was tested in vivo using a xenograft model of pancreatic cancer tumors in immunosuppressed mice. As a first proof-of-principle, the anti-tumor effect of HCA was demonstrated in vivo by reducing tumor volumes by 56% on average, even causing some tumors to disappear. The reduction in tumor volume was greater with gemcitabine, 64% on average, although gemcitabine alone did not produce the complete disappearance of any tumor in the mice, and it is also known that resistance to this drug may develop [41]. The combination of HCA and gemcitabine displayed greater efficacy, reducing tumor volumes by 90% on average and making tumors disappear in some mice. Combining drugs to fight cancer is now a widely used strategy, and different drugs are currently being combined to treat pancreatic cancer, such as FOLFIRINOX or nab-paclitaxel together with gemcitabine depending on the tumor stage [42]. Taking into account the pharmacological safety of HCA previously demonstrated in zebrafish, *Drosophila melanogaster*, and mice [11,12,13,14], and the absence of toxicity in mice shown in the present study, HCA alone or in combination with gemcitabine could represent a novel strategy to treat pancreatic cancers that deserves investigation in clinical trials.

The main antitumoral mechanism of action of HCA in pancreatic cancer is to provoke the death of pancreatic cancer cells by inducing apoptosis. When pancreatic cancer cells treated with HCA were assessed by microscopy, they exhibited membrane blebbing without any loss of membrane integrity, a characteristic of apoptotic cells [43]. An increase in the sub-G1 peak indicative of DNA fragmentation in HCA-treated cells was also observed by flow cytometry, as was proteolytic cleavage of the PARP protein. The normal function of PARP-1 is to achieve routine repair of DNA damage by adding poly (ADP ribose) polymers in response to a variety of cellular stresses, although it also participates in other cellular functions like cell death [44]. When PARP is cleaved by caspase 3 (effector caspase) or other caspases, it no longer repairs DNA and DNA fragments, leading to cell death [44]. HCA activates caspase 3 and 7 (confirmed by caspase 3 cleavage induction and luminescence Caspase 3/7 activity assay), activating the proteolytic cleavage of PARP in pancreatic cancer cells. HCA also causes cleavage of caspase 8 and 9, activating the extrinsic or death receptor-dependent pathway and the intrinsic or mitochondrial pathway of apoptosis, respectively.

The apoptosis-induced cell death triggered by HCA was reversed by inhibiting caspase 8 activity, indicating that HCA mainly triggers death in pancreatic cancer cells through the extrinsic apoptotic pathway. By contrast, inhibiting caspase 9 only produced a modest reduction in proteolytic cascade activation without impacting HCA-induced cell death. These results suggest the independent activation of both apoptosis inducer pathway and the main participation of the extrinsic pathway in antitumoral effect of HCA. Interestingly, the inhibition of both caspases 8 and 9 simultaneously did not protect against apoptosis upon HCA treatment as the caspase 8 inhibitor alone did. In that sense, the additive toxicity of both caspase inhibitors could account for the lack of prevention against HCA antitumor activity. The extrinsic pathway of apoptosis is death receptor dependent and multiple death receptors exist (e.g., TNFR or Fas), characterized by a cytoplasmic death domain that allows these receptors to initiate cytotoxic signals when is activated [17]. Apoptosis-related molecules are more likely to present in cluster in enriches lipid-raft domains in the plasma membrane, including the cell death receptor and DISC components. Moreover, capping of the Fas receptor is necessary for optimal Fas signaling in some cells, and it is ceramide-dependent [45]. Here, we observed capping of the Fas death receptor in cells exposed to HCA, probably due to the changes in the structure of membrane lipids produced by this melitherapeutic drug [39], highlighting the role of the lipid composition and structure in the modulation of membrane-associated proteins, including receptors, involved in cell survival without ligand engagement.

ROS production enhancement in pancreatic cancer cells is also provoked by HCA, leading to oxidative stress and possibly contributing to cell death. The induction of apoptosis on pancreatic cancer cells upon lipid metabolism impairment and ROS induction has been already described for tannic acid, a plant polyphenol [46]. On the other hand, the downregulation of FLIP by FAS activation can be regulated by a ROS-dependent ubiquitin-proteisimal degradation process [47]. Therefore, the participation of ROS in HCA-mediated apoptosis cannot be discarded and needs further assessment. Indeed, compounds that modulate cellular ROS can increase the death of multidrug resistance cancer cells and sensitize them to chemotherapies [48], suggesting that HCA could be a good candidate for the treatment of pancreatic cancer with current therapies like gemcitabine or FOLFIRINOX.

Considering the relevant effect of HCA in pathway related to cell stress and macroautophagy in glioblastoma cells [11], the activation status of key proteins on those pathways in pancreatic cancer cells was assessed after HCA treatment. Accordingly, HCA also activates other pathways which regulate the cell stress and, finally, the programmed cell death, the ER stress/UPR cascades by inducing BiP and CHOP protein expression and increased c-Jun phosphorylation. When physiological and pathological conditions cause ER stress in cells, the UPR is activated to restore ER homeostasis. However, when ER stress persists, the protective effect of the UPR shifts to the induction of cell death [49].

Indeed, CHOP induction has been proved to be triggered by a prolonged UPR response that would ultimately initiate cell death by apoptosis [50]. In this sense, other compounds such as gossypol induce apoptosis through the PERK-CHOP pathway in pancreatic cancer cells [51]. Previous studies have demonstrated that CHOP protein induction is critical to induce authophagy after HCA treatment in glioma cells, reinforcing its relevance to the antitumor activity of HCA [11]. Therefore, CHOP protein could be postulated as a trigger for cell death by apoptosis or autophagy depending on the cancer cell types [52]. For example, 2OHOA induces ER stress together with autophagy in glioma cells [53] or apoptosis in Jurkat cells [54]. This dual effect of a melitherapeutic drug depending on the cancer cells type is also observed with the HCA, which induces ER stress/UPR, triggering autophagy in glioma cells [11] and apoptosis in all pancreatic cancer cells studied.

HCA is incorporated into pancreatic cancer cells, modifies the composition and structure of cell membranes [13,55], and is metabolized by α-oxidation [32]. C21:5n-3 is identified as HCA’s main metabolite and can be detected in cells exposed to HCA, and not to DHA, in a time-dependent manner. Previous studies showed that HCA administration in mice produces elevated levels of C21:5n-3 in blood plasma, the brain and in glioma xenograft tumors [11,30]. Besides, the present work confirms that the metabolite does exhibits similar safety profile as the parent molecule, as shown by the Irwin test. This metabolite also has anti-proliferative activity *per se*, however, it does not seem to induce cell death by apoptosis or activate CHOP-dependent pathways. For that matter, HCA-induced cell death is apparently independent of its metabolization and C21:5n-3 production, as indicated by the absence of protection after incubating the cells with OT.

On the other hand, the treatment with both HCA and C21:5n-3 compounds trigger ER stress and the UPR pathway, as shown by the increase of c-Jun phosphorylation and BiP induction, and they enhance autophagic flux by increasing the conversion of LC3B-I to LC3B-II and SQSTM1/p62 levels [22], as already observed in glioma cells [11]. This increase in autophagic flux was partially inhibited by the α-oxidation inhibitor, OT, suggesting that formation of the C21:5n-3 metabolite is necessary to enhance autophagic flux in pancreatic cancer cells. Since OT does not inhibit the antiproliferative effect of HCA, but rather stimulates it, the metabolite production in the cell by α-oxidation and the induction of the autophagic flux due to HCA in pancreatic cancer cells seems not relevant for provoking cell death. Indeed, it is worth noting the additive effect between HCA and OT, whereby inhibiting α-oxidation, hence the metabolite formation and the autophagic flux, would potentiate the effect of HCA. Several publications have described how inhibiting autophagy flux, as OT does when combined with HCA, enhances the apoptosis death induction via CHOP [52]. For instance, the compound Edelfosine induces apoptosis and ER stress and has been shown to potentiate apoptosis induction using autophagy inhibitors in pancreatic cancer stem cells [56]. The additive antiproliferative effect of HCA and OT could be also justified by the OT antitumor effect *per se* [34], in part due to the inhibition of transketolase, a key enzyme in the pentose-phosphate pathway [57,58], despite reducing C21:5n-3 levels. In this context, it would be interesting to investigate the antitumor effect of HCA in vivo when α-oxidation is fully and specifically inhibited, which could not be reached by using OT. Further studies would be needed using Hacl KO cells or Hacl KO mice [59,60] in which α-oxidation is completely inhibited, potentially clarifying whether the full inhibition of HCA metabolization could constitute a good approach to potentiate its antitumoral activity.

Given all the above, the administration of HCA as antitumoral drug for the treatment of pancreatic cancer, inducing cancer cell death by apoptosis, has proven to be promising alone or in combination with other compounds, such as already approved chemotherapies, like gemcitabine, an α-oxidation inhibitor to enhance HCA effect.

## 4. Materials and Methods

### 4.1. Cell Cultures, Drugs, and Cell Proliferation Assays

The pancreatic cancer cell lines used here were obtained from Apointech SI (Salamanca, Spain), and the MIA PaCa-2 and PANC-1 cells were cultured in DMEM (Sigma-Aldrich, St. Louis, MO, USA), while the BxPC-3 cells were cultured in RPMI 1640 medium (Sigma-Aldrich, St. Louis, MO, USA) supplemented with 10% Fetal Bovine Serum (FBS: Sigma-Aldrich), 2 mM glutamine, 100 mM sodium pyruvate, and 350 g/L glucose. The cells were all incubated at 37 °C and in 5% CO_2_. HCA and DHA were obtained from Medalchemy (Alicante, Spain). Cell viability was determined using Trypan blue staining and metabolically active cells were analyzed using the cell proliferation kit II (Roche, Basel, Switzerland), according to the manufacturer’s instructions. These assays were performed as described previously [61], and the IC_50_ values (concentration of drug required for 50% inhibition) were obtained.

### 4.2. Animals, Tumor Xenografts, and Treatments

Male and female NUDE (Swiss) Crl:NU (Ico)-Foxn1^nu^ mice (8-weeks-old, 25–35 g: Charles River Laboratories, Paris, France) were maintained in a thermostat cabinet (28 °C: EHRET, Labor-U-Pharmatechnik, Emmendingen, Germany) with a sterile air flow at a relative humidity of 40–60% and on a 12 h dark/light cycle. Autoclaved food and water were supplied *ab libitum*. To instigate xenograft tumors, 7.5 × 10^6^ MIA PaCa-2 cells were inoculated subcutaneously into both sides of the animal’s dorsal flank and tumors became visible after two weeks. The animals were divided randomly into groups that were treated for 40 days with: the vehicle alone (ethanol 5%, p.o., daily); HCA (200 mg/kg, p.o., daily); Gemcitabine (100 mg/kg, i.p., twice weekly), a combination of HCA (100 mg/kg, p.o., daily) and Gemcitabine (100 mg/kg, i.p., once weekly); or C21:5n-3 (200 mg/kg, p.o., daily). Tumor volumes (v) were calculated as v = W^2^ × L/2, where W is the tumor width and L its length. Finally, animals were euthanized, and their tumors were analyzed histologically. Briefly, tumors were fixed overnight in 10% paraformaldehyde, dehydrated, and embedded in paraffin. Serial tissue sections were then stained with either hematoxylin and eosin for histopathological evaluation, or Ki-67 to assess proliferation (Ready to Use, Agilent Technologies-DAKO, Santa Clara, CA, USA). Toxicity was studied based on the Irwin test [35] after an acute dose of C21:5n-3 (or vehicle, controls) was administered to 4-month-old CD-1 mice (*n* = 3 each, see Supplementary Figure S4). All experiments were carried out in accordance with the animal welfare guidelines of the European Union (Directive 2010/63/EU) and the regional government of the Balearic Islands (Conselleria d’Agricultura, Pesca I Alimentació, code: 2018/09/AEXP, approved 08-10-2018).

### 4.3. Cell Cycle Analysis, Commercial Inhibitors, and Caspase-3/7 Activity

The cell cycle was analyzed as described previously [62]. Ros scavengers used were: α-tocopherol (100 µM, Vit. E) or Ferrostatin-1 (1 µM). The commercial inhibitors used were: Z-IETD-FMZ (50 µM: Selleckchem, Houston, TX, USA), Z-LEHD-FMK (50 µM: Selleckchem, Houston, TX, USA) and Oxythiamine chloride hydrochloride (OT, 1 and 10 mM: Santa Cruz Biotechnology, Heidelberg, Germany). The cells were seeded in culture plates and pre-treated with commercial inhibitors for 1.5 h before they were exposed to HCA for different times. Caspase-3/7 activity was measured using the Caspase-Glo^®^ 3/7 assay according to the manufacturer’s instructions (Promega, Madison, WI, USA).

### 4.4. Cell Lysis, Protein Quantification, Electrophoresis, and Immunoblotting

Cells were washed with PBS and scraped off the plates in 200 μL of protein extraction buffer (10 mM Tris-HCl, 2 mM EDTA, 1% SDS, 5 mM Complete Protease Inhibitor cocktail (Roche), and 1 mM Sodium Orthovanadate). The protein extract was quantified and immunoblotting was performed as described elsewhere [31], with minimal modifications. After resolving the extracts by electrophoresis and transferring them to membranes, they were probed overnight with primary antibodies (diluted in fresh blocking solution) raised against: BiP, Caspase 8, Caspase 9, CHOP, p-c-Jun (Ser63), and LC3B, (1:1000 dilution: Cell Signaling, Leiden, The Netherlands), Caspase 3, p-cJun, and PARP (1:1000 dilution: Santa Cruz Biotechnology, CA, USA); SQSTM1/p62 (1:5000 dilution: Thermo-Scientific/Pierce, Barcelona, Spain). Antibody binding was detected by incubating the membranes for 1 h at room temperature with IRDye (800 CW) conjugated anti-mouse or anti-rabbit IgG (Li-cor, Biosciences, Lincoln, NE, USA) diluted 1:5,000 in blocking solution. Finally, the labelling was quantified by integrated photodensitometry after near infrared scanning at 700 nm (Odissey, Li-cor Biosciences, Lincoln, NE, USA) and using α-Tubulin (1:10,000 dilution: Sigma) as a loading control.

### 4.5. Immunofluorescence and Confocal Microscopy

Cells were seeded onto 25 mm circular coverslips in 24-well plates at a density of ca. 15,000 cells/well. The cells were exposed to HCA for 48 h, fixed and immunostained as described previously [53]. In some cases, Wheat Germ Agglutinin (WGA) Alexa Fluor™ 594 Conjugate was used (Invitrogen; Thermo Fisher Scientific, Inc., Waltham, MA, USA). To study the generation of ROS species, cells were cultured in the presence or absence of HCA for 48 h, and they were then incubated with dichlorofluorescein diacetate (10 µM) for 30 min at 37 °C.

### 4.6. Fatty Acid Analysis by Gas Chromatography (GC)

Pancreatic cancer cell cells were seeded in 10 cm diameter culture plates at a density of 1 × 10^6^ cells/plate and they were maintained in the presence or absence of the compounds for different periods of time. Lipids were extracted directly from the cell lysates, as described previously [31].

### 4.7. Data Analysis

The data were expressed as the mean ± SEM or as the % relative to the untreated controls from independent experiments, as indicated in each figure. The significance of the differences was assessed with a Student’s *t*-test (two groups) or a one-way ANOVA (comparison between several groups: GraphPad Prism 6.1). The level of significance was set at a 95% level of confidence (*p* < 0.05).

## 5. Conclusions

HCA is a hydroxylated DHA derivative that exerts an anti-proliferative effect against cellular and murine models of human pancreatic cancer cells, inducing apoptosis and ER stress, and triggering an increase in ROS species. HCA is a molecule developed in the context of melitherapy and it is metabolized through α-oxidation to the fatty acid C21:5n-3 metabolite, which also inhibits tumor cell proliferation. Given the medical need for improved therapies for pancreatic cancer, HCA represents a promising and safe innovative anticancer drug candidate to be administrated alone or in combination with other drugs, like gemcitabine.

## Data Availability

All the data generated or analyzed in this study are included in the manuscript.

## Supplementary Materials

The supporting information can be downloaded at: https://www.mdpi.com/article/10.3390/ijms23179902/s1.
