# Supplementary material for: HCA (2-Hydroxy-Docosahexaenoic Acid) Induces Apoptosis and Endoplasmic Reticulum Stress in Pancreatic Cancer Cells
Source: Int J Mol Sci. 2022 Aug 31;23(17):9902. doi: 10.3390/ijms23179902 (PMC9456069; doi:10.3390/ijms23179902)
Supplement: Supplementary file 1 [file ijms-23-09902-s001.zip › ijms-1825660-supplementary.pdf]

SUPPLEMENTARY TABLE

Table S1. Efficacy of HCA, gemcitabine, and the combination of HCA and gemcitabine against different pancreatic cancer cell lines. The IC_{50} values for HCA, gemcitabine, and the combination of HCA and gemcitabine against pancreatic cancer cell proliferation after a 48 h treatment (mean ± SEM from 3 independent experiments performed in quadruplet). HCA, 2-hydroxycervonic acid.

Cell line	Treatment 48 h	IC_{50}
MIA PaCa-2	Gemcitabine	55 ± 21 nM
	HCA	205 ± 05 μM
	Gemcitabine + HCA 175 μM	2 ± 1.9 nM
	HCA + Gemcitabine 25 nM	145 ± 10 μM
BxPC-3	Gemcitabine	180 ± 25 nM
	HCA	218 ± 06 μM
	Gemcitabine + HCA 175 μM	93 ± 40 nM
	HCA + Gemcitabine 25 nM	199 ± 02 μM
PANC-1	Gemcitabine	–
	HCA	232 ± 16 μM

SUPPLEMENTARY FIGURES

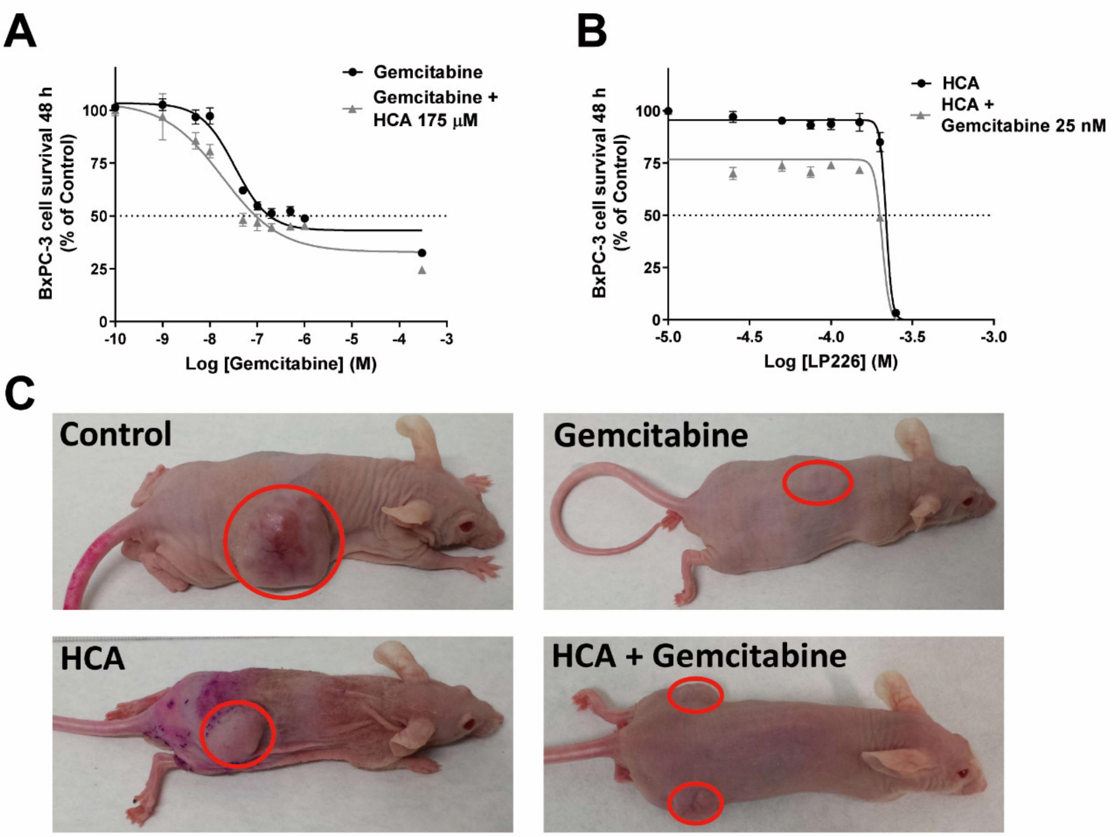

D

Groups	Animal no.	Differentiation	Pattern	Necrosis %	Mitosis/10 HPFs*	Leuco. Infiltration
Control	B1	Poor	Diffuse	20%	9	Grade 3
	B2	Poor	Diffuse	20%	14	Grade 3
	B3	Poor	Diffuse	20%	6	Grade 3
	B4	Poor	Diffuse	20%	11	Grade 3
HCA	B12	Poor	Diffuse	20%	11	Grade 3
	B13	Poor	Diffuse	20%	12	Grade 3
	B14	Poor	Diffuse	20%	7	Grade 3
	B15	Poor	Diffuse	20%	8	Grade 3

*Mitotic count per 10 high-power fields.

Figure S1. Efficacy of HCA to combat pancreatic cancer. (A) Concentration-dependent inhibition of human pancreatic cell (BxPC-3) cell growth by gemcitabine and gemcitabine in combination with HCA (175 μM) for 48 h. (B) Concentration-dependent inhibition of human pancreatic cell (MIA PaCa-2) growth by HCA and HCA in combination with gemcitabine (25 nM) for 48 h (mean ± SEM from 3 independent experiments performed in quadruplet). (C) Representative photographs of mice that received a xenograft of MIA PaCa-2 cells and treated with the vehicle alone (Control), gemcitabine (100 mg/kg, ip, twice weekly), HCA (200 mg/kg, p.o., daily), or HCA + gemcitabine (100 mg/kg, p.o., daily + 100 mg/kg, i.p., once weekly) over a 40-day treatment. (D) Analysis of histopathological images of tumors. Grading used at finding level: Grade 1 (minimal/very few/very

small), grade 2 (slight/few/small), grade 3 (moderate/moderate number/moderate size), grade 4 (marked/many/large), grade 5 (severe/massive). HCA, 2-hydroxycervonic acid.

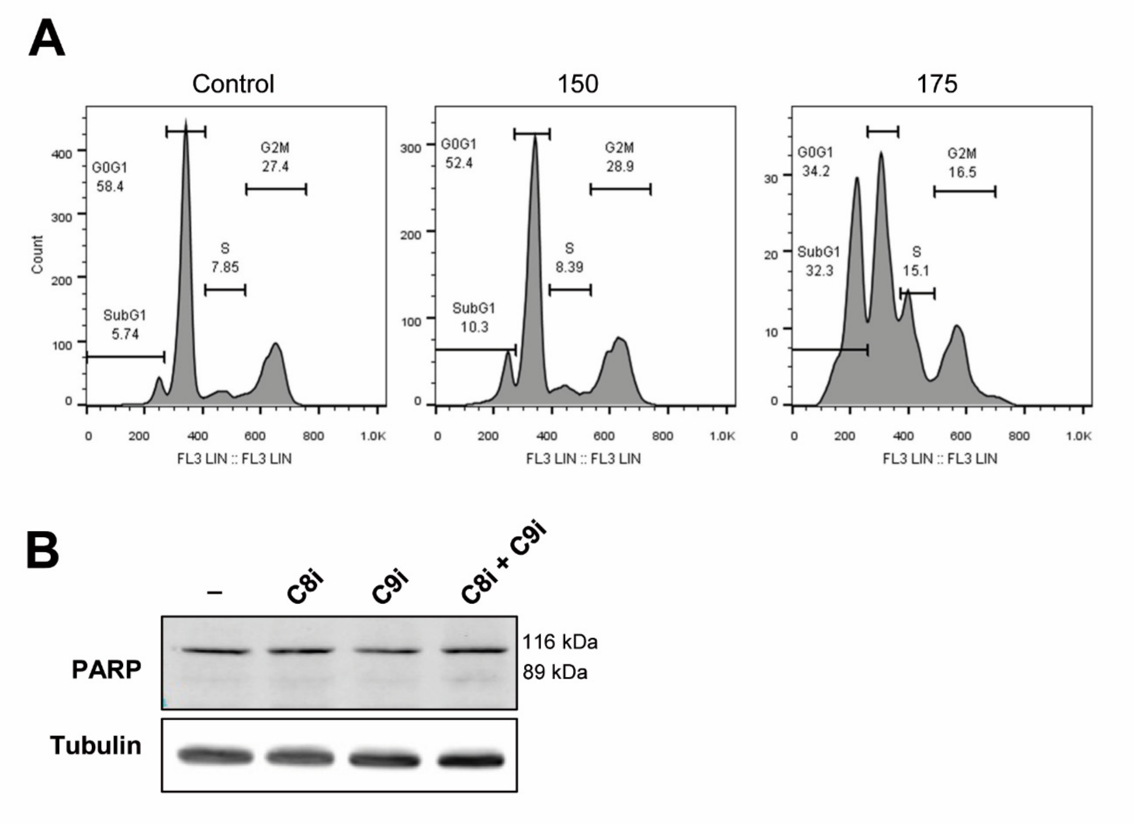

Figure S2. Figure 2. HCA induces intrinsic and extrinsic apoptosis in pancreatic cancer cells. (A) Representative histogram of the cell cycle phases at different phases of the cell cycle: distribution of MIA PaCa-2 cells incubated with or without HCA (150 or 175 µM) for 48 h. (B) Representative immunoblot of the effects of C8i (inhibitor zIETD-fmk, 50 µM), C9i (inhibitor zLEHD-fmk, 50 µM) and C8i+C9i (zIETD-fmk+zLEHD-fmk, 50 + 50 µM) on PARP proteolysis in MIA PaCa-2 cells for 48 h. C8i, caspase 8 inhibitor (zIETD-fmk); C9i, caspase 9 inhibitor (zLEHD-fmk); C8i+C9i, caspase 8 inhibitor + caspase 9 inhibitor (zIETD-fmk+zLEHD-fmk); PARP, Poly (ADP-Ribose) Polymerase.

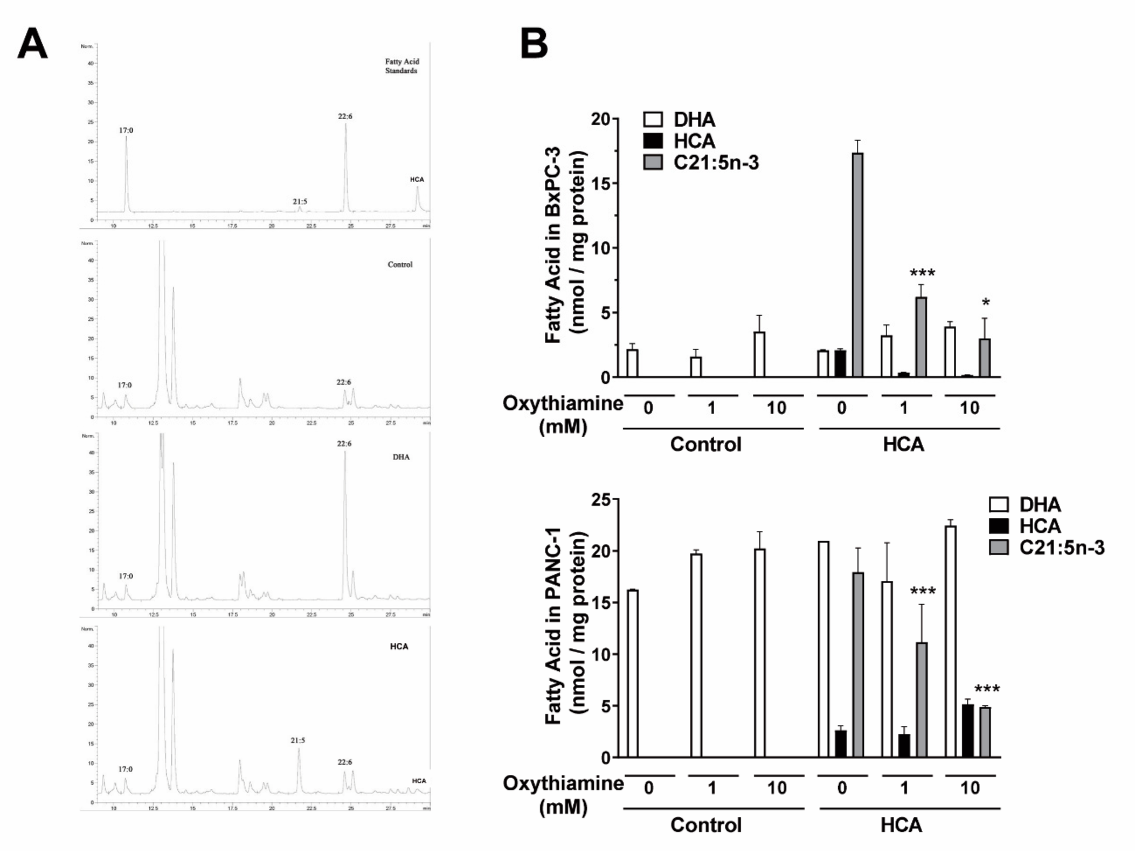

Figure S3. Effect of HCA on the fatty acid composition of pancreatic cancer cells. (A) Amplified regions of representative GC-FID chromatograms showing the fatty acid composition following different treatments of MIA PaCa-2 cells: fatty acid standards, control (untreated, 48 h), DHA (100 µM, 48 h) and HCA (150 µM, 48 h). The peaks of interest were identified with standards based on their retention time. (B) BxPC-3 and PANC-1 cells were pretreated with oxythiamine (OT, 1 or 10 mM) for 1.5 h before exposure to HCA (150 µM) for 48 h, and the cell's lipids were extracted and quantified by GC (bars correspond to the mean ± SEM values of 3 independent experiments). HCA, 2-hydroxycervonic acid; DHA, docosahexaenoic acid; C21:5n-3, heneicosapentaenoic acid. * $p < 0.05$, *** $p < 0.001$

	Control	C21:5n-3					
		50 mg/kg	100 mg/kg	200 mg/kg	400 mg/kg	800 mg/kg	1500 mg/kg
**Physical factors and gross appearance**							
Body weight (g) 1 h	31.7 ± 5.6	38.4 ± 0.4	38.8 ± 3.5	38.2 ± 3.3	41.3 ± 5.4	42.4 ± 4.6	39.2 ± 9.3
Body weight (g) 24 h	31.2 ± 5.3	36.5 ± 1.2	37.5 ± 3.5	37.5 ± 3.3	41.9 ± 5.5	38.4 ± 4.1	37.0 ± 9.6
Presence of whiskers	3.0 ± 0.0	3.0 ± 0.0	3.0 ± 0.0	3.0 ± 0.0	3.0 ± 0.0	3.0 ± 0.0	3.0 ± 0.0
Appearance of fur	2.0 ± 0.0	2.0 ± 0.0	2.0 ± 0.0	2.0 ± 0.0	2.0 ± 0.0	2.0 ± 0.0	2.0 ± 0.0
Patches of missing fur on face	0.0 ± 0.0	0.0 ± 0.0	0.0 ± 0.0	0.0 ± 0.0	0.0 ± 0.0	0.0 ± 0.0	0.0 ± 0.0
Patches of missing fur on body	0.0 ± 0.0	0.0 ± 0.0	0.0 ± 0.0	0.0 ± 0.0	0.0 ± 0.0	0.0 ± 0.0	0.0 ± 0.0
Wounds	0.0 ± 0.0	0.0 ± 0.0	0.0 ± 0.0	0.0 ± 0.0	0.0 ± 0.0	0.0 ± 0.0	0.0 ± 0.0
**Observation of behavior in a novel environment**							
Transfer behavior	5.0 ± 0.0	4.7 ± 0.6	5.0 ± 0.0	4.3 ± 0.6	4.7 ± 0.6	5.0 ± 0.0	4.8 ± 0.5
Body position	3.0 ± 0.0	3.0 ± 0.0	3.0 ± 0.0	3.0 ± 0.0	3.0 ± 0.0	3.0 ± 0.0	3.0 ± 0.0
Spontaneous activity	2.0 ± 0.0	1.3 ± 0.6	1.3 ± 0.6	1.0 ± 0.0	1.3 ± 0.6	1.3 ± 0.6	1.3 ± 0.5
Respiration rate	2.0 ± 0.0	2.0 ± 0.0	2.0 ± 0.0	2.0 ± 0.0	2.0 ± 0.0	2.0 ± 0.0	2.0 ± 0.0
Tremor	0.0 ± 0.0	0.0 ± 0.0	0.0 ± 0.0	0.3 ± 0.6	0.0 ± 0.0	0.0 ± 0.0	0.3 ± 0.5
Palpebral closure	0.0 ± 0.0	0.0 ± 0.0	0.0 ± 0.0	0.0 ± 0.0	0.0 ± 0.0	0.0 ± 0.0	0.0 ± 0.0
Piloerection	0.0 ± 0.0	0.0 ± 0.0	0.0 ± 0.0	0.0 ± 0.0	0.0 ± 0.0	0.0 ± 0.0	0.0 ± 0.0
Gait	0.0 ± 0.0	0.0 ± 0.0	0.0 ± 0.0	0.0 ± 0.0	0.0 ± 0.0	0.0 ± 0.0	0.0 ± 0.0
Tail elevation	1.0 ± 0.0	1.0 ± 0.0	1.0 ± 0.0	1.0 ± 0.0	1.0 ± 0.0	1.0 ± 0.0	1.0 ± 0.0
Urination	0.0 ± 0.0	0.3 ± 0.6	0.0 ± 0.0	0.3 ± 0.6	0.3 ± 0.6	0.7 ± 0.6	0.5 ± 0.6
Defecation	2.5 ± 0.7	1.3 ± 1.5	0.3 ± 0.6	0.7 ± 1.2	1.0 ± 1.7	0.3 ± 0.6	0.0 ± 0.0
**Reflexes and reactions to simple stimuli**							
Touch escape	3.0 ± 0.0	3.0 ± 0.0	3.0 ± 0.0	3.0 ± 0.0	3.0 ± 0.0	3.0 ± 0.0	3.0 ± 0.0
Positional passivity	1.0 ± 0.0	1.0 ± 0.0	1.0 ± 0.0	1.0 ± 0.0	0.7 ± 0.6	0.3 ± 0.6	0.5 ± 0.6
Trunk curl	1.0 ± 0.0	1.0 ± 0.0	1.0 ± 0.0	1.0 ± 0.0	1.0 ± 0.0	1.0 ± 0.0	1.0 ± 0.0
Reaching reflex	3.0 ± 0.0	3.0 ± 0.0	3.0 ± 0.0	3.0 ± 0.0	3.0 ± 0.0	3.0 ± 0.0	3.0 ± 0.0
Body tone	1.0 ± 0.0	1.0 ± 0.0	1.0 ± 0.0	1.0 ± 0.0	1.0 ± 0.0	1.0 ± 0.0	1.0 ± 0.0
**Measures recorded during supine restraint**							
Plantar surface skin color	1.0 ± 0.0	1.0 ± 0.0	1.0 ± 0.0	1.0 ± 0.0	1.0 ± 0.0	1.0 ± 0.0	1.0 ± 0.0
Heart rate	1.0 ± 0.0	1.0 ± 0.0	1.0 ± 0.0	1.0 ± 0.0	1.0 ± 0.0	1.0 ± 0.0	1.0 ± 0.0
Limb tone	2.0 ± 0.0	2.0 ± 0.0	2.0 ± 0.0	2.0 ± 0.0	2.0 ± 0.0	2.0 ± 0.0	2.0 ± 0.0
Abdominal tone	1.0 ± 0.0	1.0 ± 0.0	1.0 ± 0.0	1.0 ± 0.0	1.0 ± 0.0	1.0 ± 0.0	1.0 ± 0.0
Righting reflex	0.0 ± 0.0	0.0 ± 0.0	0.0 ± 0.0	0.0 ± 0.0	0.0 ± 0.0	0.0 ± 0.0	0.0 ± 0.0
Air Righting reflex	0.0 ± 0.0	0.0 ± 0.0	0.0 ± 0.0	0.0 ± 0.0	0.0 ± 0.0	0.0 ± 0.0	0.0 ± 0.0
Salivation	0.0 ± 0.0	0.0 ± 0.0	0.0 ± 0.0	0.0 ± 0.0	0.0 ± 0.0	0.0 ± 0.0	0.0 ± 0.0
Provoked Biting	1.0 ± 0.0	1.0 ± 0.0	1.0 ± 0.0	1.0 ± 0.0	1.0 ± 0.0	0.7 ± 0.6	1.0 ± 0.0
**Motor coordination**							
Grip strength	3.0 ± 0.0	3.0 ± 0.0	3.0 ± 0.0	3.0 ± 0.0	3.0 ± 0.0	3.0 ± 0.0	3.0 ± 0.0
Wire maneuver	0.0 ± 0.0	0.3 ± 0.6	0.0 ± 0.0	0.3 ± 0.6	0.3 ± 0.6	0.7 ± 0.6	1.0 ± 0.0
Inverted screen (s)	60.0 ± 0.0	53.3 ± 11.5	58.3 ± 2.9	60.0 ± 0.0	60.0 ± 0.0	58.3 ± 2.9	49.0 ± 12.2
Pole climb	1.5 ± 0.7	0.3 ± 0.6	0.3 ± 0.6	1.0 ± 1.7	0.7 ± 1.2	0.0 ± 0.0	0.8 ± 1.5

Physical factors and gross appearance

Body weight	Animal weight (g)
Presence of whiskers	0 = None; 1 = A few; 2 = Most, but not a full set; 3 = A full set
Appearance of fur	0 = Ungroomed and disheveled; 1 = Somewhat disheveled; 2 = Well-groomed (normal)
Piloerection	0 = None; 1 = Most hairs standing on end
Patches of missing fur on face	0 = None; 1 = Some; 2 = Extensive Patches
Patches of missing fur on body	0 = None; 1 = Some; 2 = Extensive
Wounds	0 = None; 1 = Signs of previous wounding; 2 = Slight wounds present; 3 = Moderate wounds present; 4 = Extensive wounds present

Observation of behavior in a novel environment

Transfer behavior	0 = Coma; 1 = Prolonged freeze (>10 sec.), then slight movement = Extended freeze, then moderate movement; 3 = Brief freeze (a few seconds), then active movement; 4 = Momentary freeze, then swift movement; 5 = No freeze, immediate movement; 6 = Extremely excited ("manic")
Body position	0 = Completely flat (on stomach); 1 = Lying on side; 2 = Lying on back; 3 = Sitting or standing; 4 = Rearing on hind legs; 5 = Repeated vertical leaping
Spontaneous activity	0 = None, resting; 1 = Casual scratch, groom, slow movement; 2 = Vigorous scratch, groom, moderate movement; 3 = Vigorous, rapid/dart movement; 4 = Extremely vigorous, rapid/dart movement
Respiration rate	0 = Gasping, irregular; 1 = Slow, shallow; 2 = Normal; Hyperventilation
Tremor	0 = None; 1 = Mild; 2 = Marked
Palpebral closure	0 = Eyes wide open; 1 = Eyes 1/2 closed; 2 = Eyes closed
Piloerection	0 = None; 1 = Coat stood on end
Gait	0 = Normal; 1 = Fluid but abnormal; 2 = Limited movement only; 3 = Incapacity
Pelvic elevation	0 = Markedly flattened; 1 = Barely touches; 2 = Normal (3 mm elevation) 3 = Elevated (more than 3 mm elevation)
Tail elevation	0 = Dragging; 1 = Horizontally extended; 2 = Elevated (Straub tail)
Urination	0 = None; 1 = Little; 2 = Moderate amount; 3 = Extensive
Defecation	Count the number of fecal boli emitted during the 3-min. period

Reflexes and reactions to simple stimuli

Touch escape	0 = No response; 1 = Mild (escape response to firm stroke); 2 = Moderate (rapid response to light stroke); 3 = Vigorous (escape response to approach)
Positional passivity	0 = Struggles when restrained by tail; 1 = Struggles when restrained b neck (finger grip, not scruffed); 2 = Struggles when held supine (on back); 3 = Struggles when restrained by hind legs; 4 = Does not struggle
Trunk curl	0 = Absent; 1 = Present
Reaching reflex	0 = None; 1 = Upon nose contact; 2 = Upon vibrasse contact; 3 = Before vibrasse contact (18 mm); 4 = Early vigorous extension (25 mm)
Body tone	0 = Flaccid, no return of cavity to normal; 1 = Slight resistance; 2 = Extreme resistance, board like

Measures recorded during supine restraint

Plantar surface skin color	0 = Blanched; 1 = Pink; 2 = Bright, deep red flush
Heart Rate	0 = Slow, bradycardia; 1 = Normal; 2 = Fast, tachycardia
Limb Tone	0 = No resistance; 1 = Slight resistance; 2 = Moderate resistance; 3 = Marked resistance; 4 = Extreme resistance
Abdominal Tone	0 = Flaccid, no return of cavity to normal; 1 = Slight resistance; 2 = Extreme resistance, board like
Righting Reflex	0 = No impairment; 1-10 = Number of seconds required to right
Air Righting Reflex	0 = No impairment; 1-10 = Number of seconds required to right
Salivation	0 = None; 1 = Slight margin of sub-maxillary area; 2 = Wet zone entire sub-maxillary area
Provoked Biting	0 = Absent; 1 = Present

Motor coordination	
Grip strength	0 = None; 1 = Slight grip, semi-effective; 2 = Moderate grip, effective; 3 = Active grip, effective; 4 = Unusually effective
Wire maneuver	0 = Active grip with hindlegs; 1 = Difficulty to grasp with hindlegs; 2 = Unable to grasp with hindlegs; 3 = Unable to lift hindlegs, falls within seconds: 4 = Falls immediately
Inverted screen (s)	0-60 = Number of seconds before falling
Pole climb	0 = Climbs down within 30 sec; 1 = Turns and climbs up the pole; 2 = Turns but the freezes; 3 = Does not move within 30 seconds or climbs down but not off the pole; 4 = Falls off

Figure S4. Toxicity study based on the Irwin test in C21:5n-3 treated mice. The Irwin test was assessed as described previously and according to the procedure table (parameters and scores). The Irwin test was evaluated after an acute dose of C21:5n-3 (or vehicle, controls) that did not produce any evidence of toxicity.
